# Overexpression of Glycerol-3-Phosphate Acyltransferase from *Suaeda salsa* Improves Salt Tolerance in Arabidopsis

**DOI:** 10.3389/fpls.2017.01337

**Published:** 2017-08-02

**Authors:** Na Sui, Shanshan Tian, Wenqing Wang, Mingjie Wang, Hai Fan

**Affiliations:** Shandong Provincial Key Laboratory of Plant Stress, College of Life Science, Shandong Normal University Jinan, China

**Keywords:** *Suaeda salsa*, glycerol-3-phosphate acyltransferase, salt stress, PG, unsaturated fatty acids, seedling

## Abstract

Glycerol-3-phosphate acyltransferase is the first acyl esterifying enzyme in phosphatidylglycerol (PG) synthesis process. In this study, we isolated and characterized the glycerol-3-phosphate acyltransferase (GPAT) gene from *Suaeda salsa* (*S. salsa*) and obtained the full length of the GPAT gene from *S. salsa* (*SsGPAT*) by 5′ and 3′ RACE. The clone contained an open reading frame (ORF) of 1167 bp nucleotides that comprised of 388 amino acid residues. Real-time PCR revealed that the mRNA accumulation of *GPAT* in *S. salsa* was induced by salt stress. The highest expression levels were observed when *S. salsa* leaves were exposed to 300 mM NaCl treatment. At the germination stage, the germination rate and root length of overexpressed Arabidopsis strains were significantly higher than WT under different concentrations of NaCl treatments, while the inhibitory effect was significantly severe in T-DNA insertion mutant strains. In the seedling stage, chlorophyll content, the photochemical efficiency of PSII, PSI oxidoreductive activity (ΔI/Io), and the unsaturated fatty acid content of PG decreased less in overexpressed strains and more in mutant strains than that in WT under salt stress. These results suggest that the overexpression of *SsGPAT* in Arabidopsis enhances salt tolerance and alleviates the photoinhibition of PSII and PSI under salt stress by improving the unsaturated fatty acid content of PG.

## Introduction

Salt stress is one of the main abiotic stresses. Soil salinization is a worldwide problem (Zhu, 2001) and limits the production of various crops all over the world (Munns and Tester, 2008). How plants sense stress signals and adapt to adverse environments are fundamental biological questions (Zhu, 2016). Throughout evolution, plants have developed many mechanisms to adjust to salt stress, such as causing a series of physiological and biochemical changes, and inducing the expression of functional and regulatory genes. The mechanism of salt stress to plant survival and resistance is complex, and requires further exploration.

Salt stress is known to inhibit photosynthesis through the process of photoinhibition. It is reported that Photosystem II (PSII) plays an important role in the process of leaf photosynthesis adapting to environmental perturbations (Baker, 1991). NaCl stress modifies PSII photochemistry in light-adapted leaves. Salt stress can reduce the activity of the PSII reaction center on the thylakoid membrane and inhibit photochemical efficiency. With the increase in salt concentration, the electron transfer rate of PSII significantly decreases. The reason might be that the function of the oxygen-evolving complex of the PSII oxidation side is damaged which lead to the reduction of electronic quantity supplied to PSII reaction center (Sharkey and Badger, 1982); on the other hand, the electronic transport of QA to QB on the PSII reductive side may be blocked. Studies on green algae cells have indicated that salt stress affects the release of oxygen from the oxygen-evolving complex of PSII (Allakhverdiev et al., 2000). Ioannidis et al. (2006) argues that NaCl stress affects charge separation of PSII and the pigment complex on the thylakoid membrane. The study conducted by Shu et al. (2010) revealed that the inhibition of PSII activity in cucumber seedlings is mainly on the side of the receptor, which prevents the electrons from QA to QB. The effect of salt stress on PSII need to be further studied.

When plants are subjected to salt stress, they develop a complex defense system that includes ion homeostasis, osmolyte biosynthesis, intracellular compartment of toxic ions and the scavenging systems of reactive oxygen species (ROS) (Hasegawa et al., 2000; Mittova et al., 2004; Stepien and Klobus, 2005; Flowers and Colmer, 2008). The cell membrane regulates the transport of most ions and the large molecules as a barrier in plants. The permeability of the membrane is sensitive to salt stress. It has been reported that under 100 mM NaCl stress, the membrane permeability of soybean seedlings significantly increased, and the biological function of the membrane was destroyed (Wei and Chen, 2000). Membrane fluidity can affect ATPase activity (Cooke and Burden, 1990; Liu et al., 2008), bilayer permeability (Schuler et al., 1991), and carrier-mediated transport (Deuticke and Haest, 1987), while membrane fluidity and structure are affected by membrane lipid composition and the desaturation degree of fatty acid (Mikami and Murata, 2003). Many earlier studies have reported that changes in the unsaturated fatty acid content can change plant tolerance to different conditions such as cold, drought, heat, and salt (Dakhma et al., 1995; Olsson, 1995; Matos et al., 2002; Sui et al., 2007b,c; Liu et al., 2008; Sui and Han, 2014). The membrane of plants consists of lipids, proteins and sugars. In higher plants, the most abundant membrane lipids are glycolipids, which including monogalactosyl diglyceride (MGDG) and digalactosyl diglyceride (DGDG), and phospholipids including phosphatidylcholine (PC), phosphatidylethanolamine (PE), phosphatidylinositol (PI), and phosphatidylglycerol (PG). Previous study shows that lipids can protect the photosystem at salt condition. The increase of unsaturated fatty acid content of membrane lipids can protect photosystem II (PSII) and photosystem I (PSI) at both the rapid and slow phases of NaCl-induced inactivation when using wild-type and *des*A1 cells of *Synechococcus*. The unsaturation of fatty acids plays an active role in protecting the photosynthetic machinery during the slow phase (Allakhverdiev et al., 2001). Zhang et al. (2009) found that *fad6* mutant Arabidopsis seedlings that lack chloroplast fatty acid desaturase were significantly weaker on resistance to salt stress than wild-type Arabidopsis plants. PG is the site of oxygenic electron transport in PSII and also the only phospholipid of thylakoid membranes (Wada and Murata, 1998). Therefore, the changes of PG fatty acid species can affect the function of photosynthetic machinery of PSII and the activities of chloroplastic antioxidant enzymes (Sui et al., 2007c; Sun et al., 2010). And the substrate selectivity of glycerol-3-phosphate acyltransferase (GPAT) is the main factor that determines the content of *cis*-unsaturated fatty acids in PG (Roughan and Slack, 1982).

Polyunsaturated fatty acids (PUFAs) are important constituents of cell membrane lipids and play an important role in the resistance of plants to salt stress. PUFAs are synthesized from saturated fatty acids. For higher plants, the synthesis of PUFAs can be divided into eukaryotic and prokaryotic routes according to the different places of synthesis. The prokaryotic pathway refers to saturated fatty acids synthesized in chloroplasts, which are catalyzed by GPAT and monoacyl-glycerol-3-phosphate acyltransferase in plastids to be phosphatidic acid (PA), and subsequently form PG and other glycerides. The eukaryotic pathway refers to saturated fatty acids synthesized in chloroplasts that become free fatty acids through hydrolysis, and these are finally transferred to the endoplasmic reticulum and catalyzed to be PA by GPAT and monoacyl-glycerol-3-phosphate acyltransferase, forming PG and other glycerides (Joyard et al., 2010).

Glycerol-3-phosphate acyltransferase (GPAT; E.C. 2.3.1.15) is the first acyl esterifying enzyme in PG synthesis process. It transfers the acyl moiety from an acyl-coenzyme A (CoA) donor (or acyl–acyl carrier protein [ACP] in plastids) to the *sn-1* position of a glycerol-3-phosphate (G3P) molecule, yielding 1-acylglycerol-3-phosphate (or lysophosphatidic acid, LPA). The GPAT gene has been cloned in a variety of plants, such as *Lycopersicum esculentum*, *Spinacia oleracea*, *Helianthus annuus*, *Oryza sativa*, tomato, Arabidopsis, and so on. It has been found that the GPAT gene is closely related to the fertility, stress tolerance, oil content of plants and seed development (Liu et al., 2013; Payá-Milans et al., 2016). Furthermore, the GPAT gene has 10 members in Arabidopsis, which are *AtGPAT1*, *AtGPAT2*, *AtGPAT3*, *AtGPAT4*, *AtGPAT5*, *AtGPAT6*, *AtGPAT7*, *AtGPAT8*, *AtGPAT9*, and *ATS1*, respectively. *AtGPAT1* and *AtGPAT6* can affect the seed setting rate of Arabidopsis that found in *atgpat1/atgpat6* double mutants (Zheng et al., 2003); Gidda et al. (2009) found that *AtGPAT9* could regulate the oil content of Arabidopsis seeds. In addition, *in vivo* experiments have shown that when *AtGPAT* of Arabidopsis is transferred into tobacco, the content of unsaturated fatty acids in PG increases and the chilling resistance of tobacco also increases (Murata et al., 1992). Furthermore, our previous study also revealed that the overexpression of *LeGPAT* can increase chilling and salt tolerance in tomato (Sui et al., 2007a; Sun et al., 2010). Overexpression of *AtGPAT* in rice can increase the content of unsaturated fatty acids in PG, and then improves the photosynthetic rates and growth at low temperature (Ariizumi et al., 2002).

*Suaeda salsa* (*S. salsa*) is an annual herbaceous succulent euhalophyte that has tolerance to salt (Song et al., 2008), drought (Huang et al., 2008), waterlogging (Song, 2009), and high health care (Zhao et al., 2002). Planting *S. salsa* can also significantly reduce the soil salinity and increase the content of soil organic matter (Zhao et al., 2002). The study of *S. salsa* has good economic and ecological benefits. *S. salsa* is native to saline soils in which the optimal NaCl concentration for plant growth is 200 mM, and 400 mM NaCl does not decrease the growth of this species (Song et al., 2009). Furthermore, *S. salsa* has demonstrated high resistance not only to salinity stress, but also to photoinhibition, even when treated with salt concentrations of as high as 400 mM NaCl with exposure to full sunlight (Lu et al., 2002). At present, halophytic species are widely being studied, especially by Chinese researchers, due to its important economical and ecological value in developing saline agriculture; and it has been used as a promising model halophyte for understanding salt tolerance. However, it remains unknown whether the overexpression of *S. salsa GPAT* in Arabidopsis can increase the unsaturated fatty acids content of PG, and whether it is relative to PSII and PSI photoprotection under salt stress. In the present study, we isolated the GPAT gene from *S. salsa* by 5′ and 3′ RACE, and transformed the gene into Arabidopsis. Our results revealed that *GPAT* from euhalophyte *S. salsa* improved salt tolerance and alleviated the salt-induced photoinhibition of PSII and PSI by increasing the unsaturated fatty acid content of PG in Arabidopsis.

## Materials and Methods

### Plant Material, Cultivation, and Treatment

Brown seeds of *S. salsa* were collected during November 2013 in the saline inland of the Yellow River Delta (N37°25′, E118°58′) in Shandong province, China. Dry seeds were stored in a refrigerator at 4°C before use.

Arabidopsis Col-0 was used as the wild-type control. Arabidopsis mutants of *gpat2* (SALK_060056) and *gpat6* (SALK_136675C) were ordered from the Arabidopsis Biological Resource Center and the homozygous mutants with a T-DNA insertion within *AtGPAT2* (At1g02390) and *AtGPAT6* (At2g38110) were verified by PCR.

The brown seeds of *S. salsa* were uniformly grown in 10 cm × 10 cm × 10 cm red square plastic pots filled with clean sand under a 14-h (28 ± 5°C)/10-h (20 ± 3°C) light/dark photoperiod, with a relative humidity of 60% ∼ 70% and an illumination intensity of 800 ± 100 μmol m^-2^ s^-1^. A total of 10 seeds were allocated for each pot. Plants were watered with complete Hoagland nutrient solution when the true leaves came out. After four weeks, some of the plants were used in the experiment to isolate *SsGPAT* and the remaining plants were treated with 0, 100, 200, 300, 400, 500, and 600 mM NaCl. The NaCl was dissolved in a nutrient solution (Hoagland nutrient solution). In the NaCl treatment, the chance of osmotic shock was reduced by adding 50 mM NaCl on the first day and the concentration was increased by 50 mM on each subsequent day until the final concentration was reached. Two weeks later, the treated leaves were then frozen in liquid nitrogen and stored at -80°C until further use for the expression pattern of the *SsGPAT* gene.

Arabidopsis seeds were sterilized by 70% ethanol for 1 min, incubated with 1% NaClO incubation for 15 min, and washed for five times with distilled water. The seeds were sown on Murashige and Skoog (MS) medium added with 0, 50, 100, and 150 mM NaCl. Then, these were stratified for three days at 4°C and transferred to the culture room at 22°C day/18°C night under a 16/8 light/dark cycle. The length of the root of the *Arabidopsis thaliana* was measured after 9 days. For adult stage experiments, the sterilized Arabidopsis seeds were uniformly plated on the MS medium, chilled at 4°C for 3 days, and then transferred to a growth room with a 16-h 25°C/8-h 25°C light/dark cycle (the relative humidity was 70%, illumination intensity was 4000 Lx). After 10 days, the seedlings were transplanted in nutrient soil, and six seedlings were planted in each 10 cm × 10 cm × 10 cm red square plastic pot and watered with 1/2 Hoagland nutrient solution. Two weeks later, the plants were treated with 1/2 Hoagland nutrient solution added with 0, 50, and 100 mM NaCl. The physiological indexes such as chlorophyll content, chlorophyll fluorescence, PSI activity and biomass were determined 14 days later.

### Cloning and Sequencing of *SsGPAT*

Total RNA was isolated from the leaves of *S. salsa* using a Total Plant RNA Extraction Kit (Karroten 1103) according to the manufacturer’s instructions. In the experiment of obtaining the intermediate fragment of the GPAT gene, the degenerate primers were designed using Primer 5 Software according to the known sequences of homologous genes in plants such as tobacco, rice, Arabidopsis and so on. The primers SsGPAT-5 and SsGPAT-3 were chosen in order for the PCR product to have approximately the same size of 440 bp. The ends sequence of *SsGPAT* was obtained by 5′ Race using a 5′ RACE Kit (5′ RACE Systerm for Rapid Amplification of cDNA Ends, Version 2.0, Invitrogen) and 3′ RACE. The primer pairs for 5′ RACE were GPAT5′-1, GPAT5′-2, GPAT5′-3, and the 3′ Race amplification were designed according to acquired intermediate fragment. The cDNA for 3′ Race amplification was reverse transcribed by QT primer instead of the primer mix and the 3′ RACE sequence was obtained through the prime pairs of Q0, GP3′-1 and Q1, GP3′-2. The full-length sequence of the *SsGPAT* was determined through the combination of the front sequencing results, and the sequence of *SsGPAT* was obtained using Gpat5′ and Gpat3′ primers. All the primer sequences are listed in **Table 1**.

**Table 1:** Primers used in this experiment.

Name	Sequence	Applicaition
SsGPAT-5	5′- CA(C/T)CA(A/G)A(G/C) TGAAGC(A/T)GATCC-3′	Intermediate fragments of *SsGPAT*
SsGPAT-3	5′- GGAGG(A/G/C)GGCAT(A/G/T)ATGTCAT(A/G)-3′	
GPAT5′-1	5′- CTCGATCACCTGCTATGTAAATC-3′	
GPAT5′-2	5′- CTGCAATGTGTGAGTTTGTCTTC-3′	5′RACE
GPAT5′-3	5′- CAAAGCAATCACGGCAGGATCTG-3′	
QT	5′-CCAGTGAGCAGAGTGACGAGGACTCGAGCTCAAGCTTTTTTTTTTTTTTTTT-3′	
Q0	5′-CCAGTGAGCAGAGTGACG-3′	
Q1	5′-GAGGACTCGAGCTCAAGC-3′	3′ RACE
GP3′-1	5′-GGTGAATGGTATCCGGCAAC-3′	
GP3′-2	5′-GAGAAGACTTGTGGAGCATG-3′	
Gpat5′	5′-ATGGCGGATGCTGCTCTTCC-3′	
Gpat3′	5′-AGGTTGTGACAAAGAGATGGTC-3′	Full length of *SsGPAT*
Actine-F	5′-GCTCTACCCCATGCAATCCT-3′	Reference sequence of *S. salsa*
Actine-R	5′-TGCTCTTGGCAGTCTCTGATT-3′	
G5	5′-CTATAAGTGTTGCTTCTG-3′	RT-PCR of *SsGPAT*
G3	5′-AATAGTCAATAGGCTCTC-3′	
35s	5′-GCAAGTGGATTGATGTGATATC-3′	Identification of overexpression lines
Gpat3′	5′-AGGTTGTGACAAAGAGATGGTC-3′	
ATactin5	5′-AAGCTGGGGTTTTATGAATGG-3′	Reference sequence of Arabidopsis
ATactin3	5′-TTGTCACACACAAGTGCATCAT-3′	
GPL 060056	5′-ACTCGCCAAGTCACAGATC-3′	Screening of homozygous mutants of Arabidopsis
GP 060056	5′-ATCTTGTGGTAGGGTTTGC-3′	
GPL 136675C	5′-CCGAGACGTTGAGCTAGTGG-3′	
GP 136675C	5′-CAAAGAAGCTGCACCAACG-3′	
LBb1	5-GCGTGGACCGCTTGCTGCAACT-3	T-DNA left border specific primer

### Bioinformatic Analysis of *SsGPAT*

BLASTp online and software such as DNAstar and DNAman were used for translating nucleic acid sequences into protein sequences, homologous sequence alignment, homology analysis. Phylogenetic tree was constructed by MEGA using Neighbor-Joining (NJ). The SMART online software was used to predict functional domains and functional classification. The MEGA software service was used for the analysis of the phylogenetic relationships of the amino acid residues of *SsGPAT* between different plants.

### Real-time PCR analysis

In order to evaluate the effects of the expression of *GPAT*, the expression profiles of the gene in the leaves of *S. salsa*, wild type Arabidopsis, the GPAT-overexpressing Arabidopsis strains and homozygous mutant Arabidopsis strains were investigated. For quantitative real-time PCR, amplification was performed with the G5 and G3 primers (**Table 1**) for the *SsGPAT* gene. The amplification of the ACTIN gene was used as an internal control, and the internal primers of Actin-F, Actin-R and ATactin5, ATactin3 (**Table 1**) were designed according to the Actin gene of *S. salsa* and Arabidopsis. The relative expression of *GPAT* was calculated by 2^-ΔΔCt^ method.

### Plasmid Construction and *Agrobacterium*-Mediated Transformation of Arabidopsis

The full-length of *SsGPAT* was inserted into plant binary vector pB7WG2D to construct pB7WG2D–*SsGPAT.* Then, the *SsGPAT* gene under the control of the CaMV35S promoter was transformed into Arabidopsis using the *Agrobacterium*-mediated inflorescences infected transformation method (Zhang X. et al., 2006).

### Isolation of the GPAT T-DNA Insertional Mutants of Arabidopsis Strains

In order to select homozygous plants, the specific primers used for *gpat2* were GPL060056 and GP060056, and the specific primers used for *gpat6* were GPL136675C, GP136675C (**Table 1**). Plants that did not generate polymerase chain reaction (PCR) products with the gene-specific primers were subsequently evaluated for the presence of the T-DNA insertion using the gene-specific forward primer and the T-DNA left border specific primer LBb1 (**Table 1**).

### Determination of Seed Germination and Root Length of Arabidopsis

The emergence of radicles from the seed coat was used as the standard of seed germination. The germination rate of WT, overexpressed lines and mutants were measured after 24 h. Germination rate (%) = number of germinated seeds/total number of seeds × 100%. The root length of these different strains was measured after 9 days.

### Lipid Extraction and Analysis

Leaves of *S. salsa* were harvested and immediately frozen in liquid nitrogen. Lipids were extracted according to the method of Siegenthaler and Eichenberger (1984). The leaves were grounded into a uniform paddle using a mortar. The paddle was transferred to a 50 ml large centrifuge tube then 3 ml chloroform and 5.4 ml KCl was added into the tube. The mixture was centrifuged at 1500 *g* for 15 min. The lower layer of the liquid was transferred into the bottom glass tube and dried with N_2_. The powder was dissolved in 0.2 ml of chloroform: methanol (2:1), and separated with two-dimensional thin layer chromatography (TLC) (Xu and Siegenthaler, 1997). For quantitative analysis, the lipids were separated by TLC, scraped from the plates, and used to prepare fatty acid methyl esters. The fatty acid composition of the individual lipids was determined using gas chromatography (GC-9A, Shimadzu, Japan) as described by Chen et al. (1994).

### Determination of Chlorophyll Content

Chlorophyll content was determined using the method described by Li et al. (2003). Arabidopsis leaves (0.2 g FW) were washed in distilled water and extracted with 80% acetone for 48 h in the dark. Absorbency at 663 and 645 were determined using a TU-1810 UV-spectrophotometer. Chlorophyll content was calculated as follows: Ca (mg/L) = 12.7A_663_ – 2.69A_645_; Cb (mg/L) = 22.9A_645_ – 4.68A_663_.

### Measurements of Chlorophyll Fluorescence

Chlorophyll (Chl) fluorescence was measured using a portable fluorometer (FMS2, Hansatech, King’s Lynn, United Kingdom) according to a previously described protocol (Kooten and Snel, 1990). Minimal fluorescence (Fo) with all PSII reaction centers open was determined with modulated light which was low enough not to induce any significant variable fluorescence (Fv) (Sui, 2015). Maximal fluorescence (Fm) with all reaction centers closed was determined by irradiating for 0.8 s with saturating light of 8,000 μmol m^-2^ s^-1^ on a dark-adapted leaf (adapted 15 min in the dark). Then the leaf was illuminated by an actinic light of 500 μmol m^-2^ s^-1^. Steady-state fluorescence (Fs) was recorded when the leaf reached steady-state photosynthesis. A second treatment with 0.8 s of saturating light of 8,000 μmol m^-2^ s^-1^ was given to determine the maximal fluorescence in the light-adapted state (Fm′) (Sui, 2015). The maximal photochemical efficiency (Fv/Fm) of PSII was expressed as Fv/Fm = (Fm–Fo)/Fm. The quantum yield of the PSII electron transport was determined using ΦPSII = (Fm′–Fs)/Fm′. Non-photochemical quenching (NPQ) and photochemical quenching (qP) were calculated as NPQ = Fm/Fm′-1 and qP = (Fm′-Fs)/(Fm-Fo′) according to Schreiber et al. (1995), respectively.

### PSI Activity

Salinity-treated Arabidopsis incubated for 30 min in the dark and PSI activity was measured using a multifunctional plant efficiency analyzer (Hansatech, MPEA-2, United Kingdom).

### Analysis of Fresh Mass and Dry Mass Per Plant of WT, Transgenic Arabidopsis Strains and T-DNA Mutants under Salt Stress

The plant material was initially cleaned with distilled water. After absorbing residual water using tissue paper, the fresh weight (FW) of the plant material was obtained. The dry weight (DW) was measured after drying the plants at 80°C for 24 h.

### Statistical Analysis

Statistical analysis is performed according to our previous method (Cheng et al., 2014). Data were transformed (arcsine) before the statistical analysis to ensure homogeneity of variance. Multiple comparisons were performed between different environmental conditions using Duncan’s test at a 0.05 significance level. All tests were performed with SPSS Version 19.0 for Windows (SPSS, Chicago, IL, United States).

## Results

### Sequence Analysis of *SsGPAT*

**FIGURE 1:**
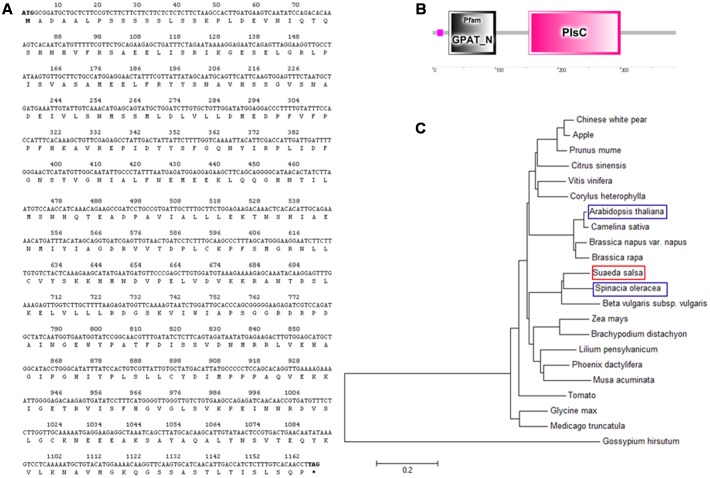
Sequence analysis of *SsGPAT*. Nucleotide sequences and amino acid residue sequences of *SsGPAT* **(A)**, structural domain prediction of the *SsGPAT* protein **(B)**; and phylogenetic relationships of amino acid residues of *SsGPAT* between different plants **(C)** are shown. The DNAman, SMART and MEGA software were used for the sequence analysis.

The SsGPAT gene contained a complete open reading frame (ORF) of 1167 bp and the SsGPAT was comprised of 388 amino acids with a molecular mass of 43 kDa (**Figure 1A**). The SsGPAT protein contained two structural domains. Highly conservative structural functional domains were found between 26 and101 amino acids. The presence of a Pls structural domain in the C-terminal indicated that the SsGPAT protein was a member of the acyltransferase family (**Figure 1B**). In order to investigate the evolutionary relationship among GPATs in plants, a phylogenetic tree of the full-length amino acid sequences was constructed using the Neighbor–Joining method. It was found that SsGPAT had the highest identities with GPAT from *Spinacia oleracea* (**Figure 1C**).

### Relative Expression Levels of *SsGPAT* in *S. salsa*

**FIGURE 2:**
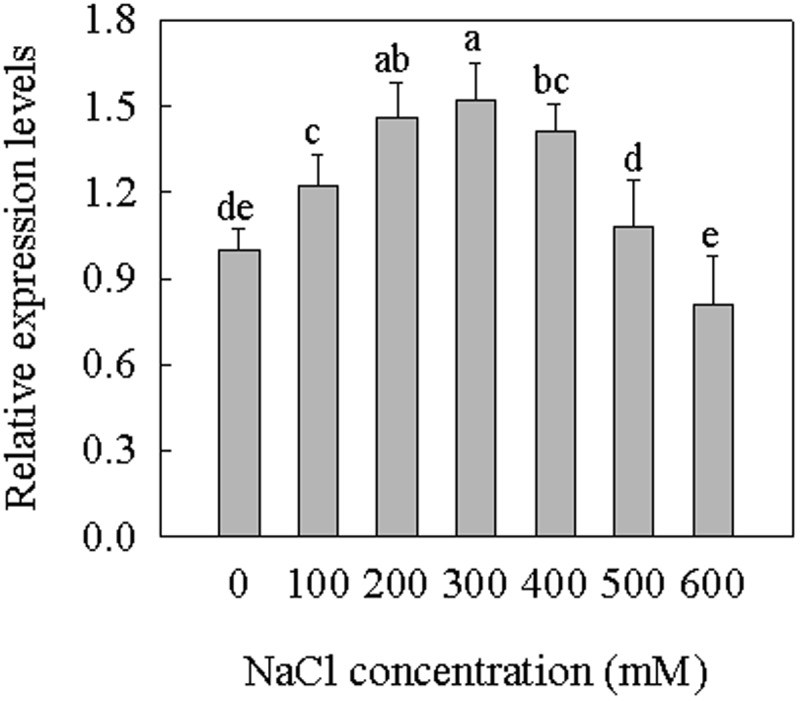
Relative expression levels of *SsGPAT* in *S. salsa*. Total RNA was isolated from leaves of seedlings in culture. The expression levels were normalized to *S. salsa* actin. Seedlings were treated with 0, 100, 200, 300, 400, 500, and 600 mM NaCl for 2 weeks. Data were expressed as means ± SD of three measurements (*n* = 3). Means identified by different letters are significantly different at *P* ≤ 0.05.

In order to determine the relative expression levels of *SsGPAT* in different salt concentrations, the accumulation of *SsGPAT* mRNA in *S. salsa* seedlings was assessed using quantitative real-time PCR (qPCR). The seedlings were watered with Hoagland nutrient solution containing 0, 100, 200, 300, 400, 500, and 600 mM NaCl. As shown in **Figure 2**, we found that the relative expression level of *SsGPAT* initially increased, reached its maximum level at 300 mM NaCl, and decreased. The expression level of *SsGPAT* at 200 and 400 mM NaCl was similar. These results revealed that 300 mM NaCl concentration is a mild salt stress for *S. salsa* which was in agreement with a study that reported that 200 mM NaCl concentration for *S. salsa* growth is optimal and 400 mM NaCl does not decrease the growth of the species (Song et al., 2009).

### Screening and Identification of the Overexpression Arabidopsis Lines

**FIGURE 3:**
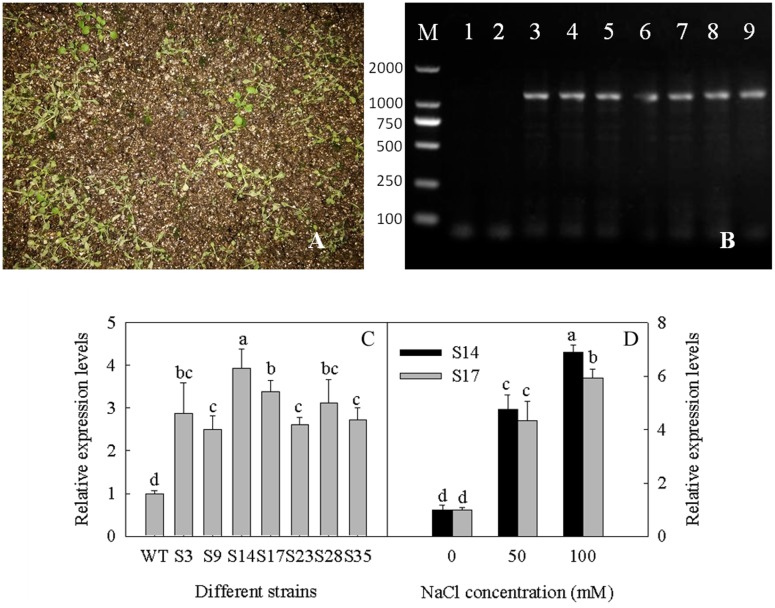
Identification of overexpressed strains. Total RNA was isolated from leaves of Arabidopsis seedlings in culture. Basta screening in overexpressing strains of Arabidopsis **(A)**; Genomic DNA PCR of overexpressed strains, lane 1 blank control, with ddH_2_O as the template; lane 2 negative control WT, with WT DNA as the template; lanes 3–9 different overexpressed strains **(B)**; The transgenic plants were tested by real time PCR, the Arabidopsis actin gene was used as a reference gene. S3-S35 indicates the different transgenic Arabidopsis strains while WT indicates the WT Arabidopsis **(C)**; Relative expression levels of *SsGPAT* in S14 and S17 when treated with 0, 50, and 100 mM NaCl **(D)**. Each column represents the means ± SD of three measurements (*n* = 3). Means identified by different letters are significantly different at *P* ≤ 0.05.

In order to understand the role of GPAT in the plant salt stress response, *SsGPAT* was overexpressed in Arabidopsis under the control of the CaMV35S promoter (**Figures 3A,B**), and seven transgenic strains that had higher expression levels of *SsGPAT* than WT were generated, including S3, S9, S14, S17, S23, S28, and S35. Given that S14 and S17 showed relatively higher expression levels of GPAT compared to other overexpression lines (**Figure 3C**) and were thus expected to exhibit better performance, we chose them for next experiments. During the treatment with 0, 50 and 100 mM NaCl, the relative expression level of *SsGPAT* in S14 and S17 significantly increased (**Figure 3D**). The reason for this might be the stability of GPAT transcript was subjected to regulation by salt stress condition, thereby protecting the plants from damage.

### Identifcation of T-DNA Insertion Mutants of *AtGPAT*

**FIGURE 4:**
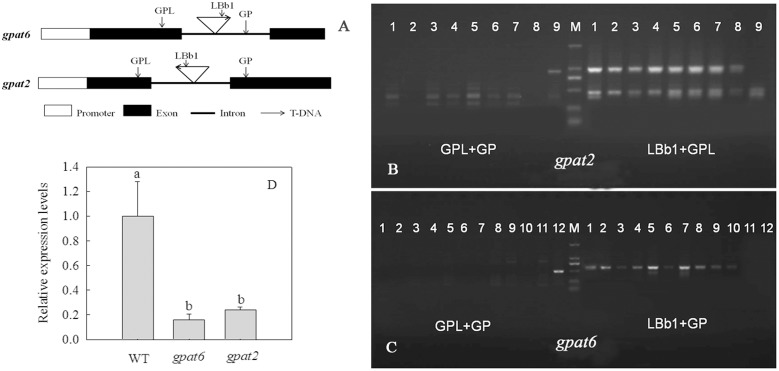
Identification of T-DNA insertion Arabidopsis mutant. The T-DNA is forward inserted at the intron of At2g38110 (*gpat6*) and reversely inserted at the intron of At1g02390 (*gpat2*) genomic locus **(A)**. T-DNA insertion was confirmed using PCR with the indicated primer sets, and the M refers to the 2000 bp marker. Lanes 1–8 *gpat2* and lane 9 WT **(B)**. Lanes 1–10 *gpat6* and lane 12 WT **(C)**. *AtGPAT* transcripts were determined by qPCR **(D)**. Data are presented as the means ± SD of three replicates (*n* = 3). Different letters indicate a significant difference at *P* ≤ 0.05.

The T-DNA were both inserted at the intron of At2g38110 (*gpat6*) and At1g02390 (*gpat2*) genomic locus and the *gpat6* is T-DNA-forward-insertion mutant, while *gpat2* is T-DNA-reverse-insertion mutant (**Figure 4A**). In order to identify the T-DNA insertion mutants, PCR analysis was performed and the amplified fragments were sequenced. Lanes 1–8 (**Figure 4B**) and lanes 1–10 (**Figure 4C**) represented homozygous Arabidopsis mutants *gpat2* and *gpat6*, respectively. *GPAT* expressed at extremely low levels in these two mutant Arabidopsis strains (**Figure 4D**). This showed that *AtGPAT2* and *AtGPAT6* have been mutant in *gpat2* and *gpat6*, respectively.

### Germination Rate and Root Length in WT, Transgenic Arabidopsis Strains and T-DNA Mutants under Salt Stress

**FIGURE 5:**
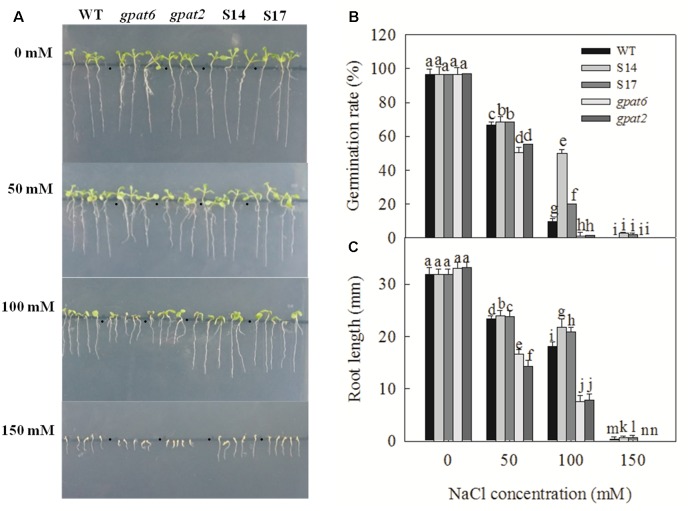
The phenotype, germination rate, root length of WT, transgenic Arabidopsis plants and T-DNA mutant Arabidopsis strains under different NaCl concentrations. The seeds were sown on Murashige and Skoog (MS) medium added with 0, 50, 100, and 150 mM NaCl and stratified for three days at 4°C before being transferred to the culture room at 22°C day/18°C night under a 16/8 light/dark cycle. The phenotype after nine days **(A)**; Germination rate after 24 h **(B)**; Root length after nine days **(C)**. Data are presented as the means of six replicates ±*SD* (*n* = 6). For each column, different letters a-n indicate a significant difference at *P* ≤ 0.05.

The germination of seeds is the basis for the growth and development of plants. Hence, the study of salt stress on seed germination is of great significance. Zhu and Hu (1996) found that the germination rate, germination index and vigor index of wheat seeds decrease under different concentration NaCl stress. In our study, there was no difference in the phenotype of wild-type, overexpressed Arabidopsis strains and mutant Arabidopsis strains without NaCl treatment (**Figure 5A**). Under NaCl treatment, the germination rate and root length of WT, the overexpressed strains and mutant Arabidopsis strains were all inhibited, and the degree of inhibition in mutant Arabidopsis strains was higher than that in WT and overexpressed strains, especially when the NaCl concentration was 100 and 150 mM (**Figures 5A–C**). During treatment with 100 mM NaCl, the germination rate of WT, S14, S17, *gpat6* and *gpat2* was 10.0, 50.0, 20.4, 1.2, and 1.9%, respectively, and was relative to the condition where NaCl treatment was not applied. When the NaCl concentration was 50 mM, the root length of WT, S14, S17, *gpat6* and *gpat2* decreased by 26.6, 24.6, 25.1%, 49.7%, and 56.9%, respectively. When the NaCl concentration was 100 mM, the root length of WT, S14, S17, *gpat6* and *gpat2* decreased by 43.3, 31.7, 34.4%, 77.1%, and 76.4%, respectively. When the NaCl concentration was 150 mM, the root length of WT, S14, S17, *gpat6* and *gpat2* decreased by 98.7, 97.8, 98.1%, 100%, and 100%, respectively. These results indicated that overexpressing GPAT decreased salt inhibition under the seed germination stage.

### Fatty Acids Composition of PG in Different Arabidopsis Lines under Salt Stress

The overexpression of *SsGPAT* increased the unsaturation of fatty acid and the double bond index (DBI = 18:1 × 1+18:2 × 2+18:3 × 3) of the major membrane lipids of PG (**Table 2**), while the mutant of GPAT decreased the unsaturated fatty acid content and DBI. In transgenic plants, a higher content of 18:2 and 18:3 was detected, while the saturated fatty acid content of 16:0 and 18:0 decreased compared to that of WT plants. The relative levels of DBI in PG increased from 80.17 in WT to 111.29 in S14 and 108.29 in S17, while DBI in PG decreased from 80.17 in WT to 76.58 in *gpat6* and 75.71 in *gpat2*. These results indicate that the overexpression of *SsGPAT* in Arabidopsis increases the content of *cis*-unsaturated fatty acids. During treatment with 100 mM NaCl, the unsaturated fatty acid content and DBI of PG all decreased in WT, transgenic plants and T-DNA mutant Arabidopsis strains. The DBI of WT, transgenic plants and the T-DNA mutant Arabidopsis strains decreased by 22.1, 14.1, 12.2, 48.6, and 45.0% under salt stress. These results showed that the synthesis of unsaturated fatty acid and DBI of PG were inhibited by salt stress, but the inhibited degree was the least in transgenic plants and was the most serious in T-DNA mutant Arabidopsis strains. This indicates that overexpression of *SsGPAT* in Arabidopsis may increase the ability to respond salt stress by improving the unsaturated fatty acid content of PG.

**Table 2:** Fatty acids composition of PG in WT, transgenic plants of S14 and S17 and T-DNA mutant Arabidopsis strains of *gpat6* and *gpat2*.

Fatty Acid (%)						

	NaCl (mM)	16: 0	16: 1	18: 0	18: 1	18: 2	18: 3	DBI
WT	0	25.01 ± 1.83^b^	34.54 ± 1.13^a^	1.56 ± 0.63^b^	13.23 ± 0.53^b^	10.01 ± 0.42^a^	15.64 ± 1.66^a^	80.17 ± 2.92^a^
	100	33.94 ± 1.11^a^	27.33 ± 1.02^b^	4.29 ± 0.31^a^	15.75 ± 0.83^a^	9.36 ± 0.53^a^	9.33 ± 1.39^b^	62.46 ± 2.61^b^
S14	0	18.64 ± 0.97^b^	27.31 ± 0.46^b^	1.42 ± 0.12^a^	15.10 ± 0.81^b^	16.43 ± 0.97^a^	21.11 ± 0.83^a^	111.29 ± 3.31^a^
	100	20.04 ± 1.45^a^	29.66 ± 0.97^a^	1.31 ± 0.69^a^	18.65 ± 0.12^a^	14.02 ± 0.44^b^	16.32 ± 0.54^b^	95.65 ± 1.02^b^
S17	0	17.74 ± 0.77^b^	26.34 ± 1.09^b^	2.69 ± 0.91^a^	17.79 ± 0.97^a^	15.82 ± 0.46^a^	19.62 ± 0.55^a^	108.29 ± 3.74^a^
	100	22.16 ± 1.45^a^	28.37 ± 3.32^a^	1.21 ± 0.35^b^	17.92 ± 0.81^a^	13.89 ± 0.31^b^	16.45 ± 1.34^b^	95.05 ± 2.47^b^
*gpat6*	0	28.36 ± 0.63^b^	34.12 ± 0.44^a^	0.86 ± 0.03^b^	9.81 ± 0.54^b^	13.81 ± 1.09^a^	13.05 ± 1.09^a^	76.58 ± 3.32^a^
	100	39.05 ± 0.91^a^	35.60 ± 0.83^a^	1.80 ± 0.42^a^	13.27 ± 0.81^a^	4.78 ± 0.54^b^	5.50 ± 0.39^b^	39.33 ± 0.56^b^
*gpat2*	0	25.20 ± 2.13^b^	36.70 ± 2.15^a^	1.88 ± 0.61^b^	10.43 ± 0.31^b^	12.09 ± 0.97^a^	13.70 ± 1.39^a^	75.71 ± 4.21^a^
	100	40.30 ± 1.83^a^	32.99 ± 0.59^b^	2.34 ± 0.41^a^	14.31 ± 0.56^a^	2.85 ± 0.19^b^	7.21 ± 0.39^b^	41.64 ± 1.44^b^

*Each point represents the mean ± SD of five measurements on each of five plants. Means identified by different letters are significantly different at *P* < 0.05*.

### Chlorophyll Content in Different Arabidopsis Lines

**FIGURE 6:**
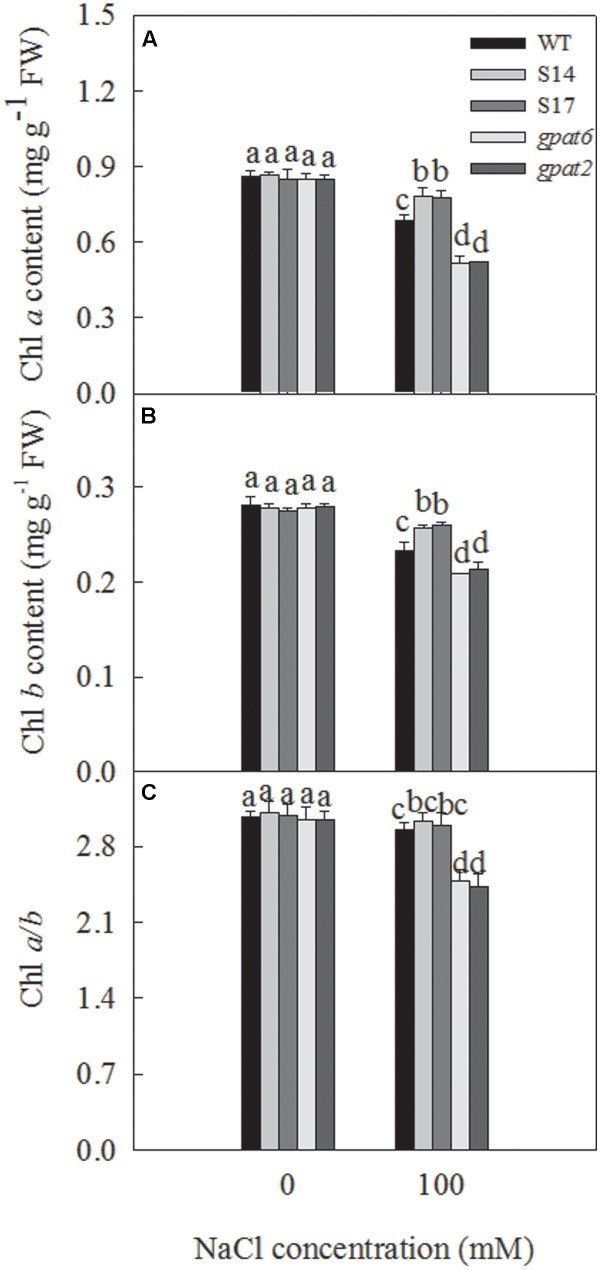
Effect of NaCl treatment on chlorophyll *a*, *b* content and *a/b* ratio of WT, transgenic Arabidopsis plants and T-DNA mutant Arabidopsis strains. The sterilized Arabidopsis seeds were uniformly plated on the Murashige and Skoog (MS) medium, chilled at 4°C for 3 days, and then transferred to a growth room with a 16-h 25°C/8-h 25°C light/dark cycle. After 10 days, the seedlings were transplanted in nutrient soil and watered with 1/2 Hoagland nutrient solution. Two weeks later, plants were treated with 1/2 Hoagland nutrient solution added with 0, 100 mM NaCl. Then, the leaves were taken for the measure of chlorophyll *a* **(A)**, chlorophyll *b* **(B)** content 14 days later, and **(C)** chlorophyll *a/b*. Data are presented as the means of six replicates ±*SD* (*n* = 6). For each column, different letters a, b, c, and d indicate a significant difference at *P* ≤ 0.05.

Chlorophyll content is directly related to the process of the photosynthetic assimilation of plants, and can be used as a physiological indicator to measure the salt tolerance of plant. Under salt stress, the chlorophyll content were reduced that affecting the growth and development of plants. Zhang R.H. et al. (2006) found that the chlorophyll content of cucumber seedlings decreases, while photosynthesis of plants is inhibited and the metabolism is disordered under salt stress. We also found that, during treatment with 100 mM NaCl, the content of chlorophyll *a*, *b* and the *a/b* ratio decreased significantly (**Figure 6**). The chlorophyll *a* content of WT, S14, S17, *gpat6* and *gpat2* decreased by 15.7, 9.5, 9.0, 39.1, and 39.0%, respectively (**Figure 6A**). The chlorophyll *b* content of WT, S14, S17, *gpat6* and *gpat2* decreased by 17.0, 8.0, 5.8, 24.9, and 23.4%, respectively (**Figure 6B**). The Chl *a/b* ratio in WT, S14, S17, *gpat6* and *gpat2* decreased from 3.07, 3.11, 3.09, 3.05 and 3.05 to 2.95, 3.04, 2.99, 2.48, and 2.43, respectively (**Figure 6C**).

### PSII and PSI Activity under Salt Stress

**FIGURE 7:**
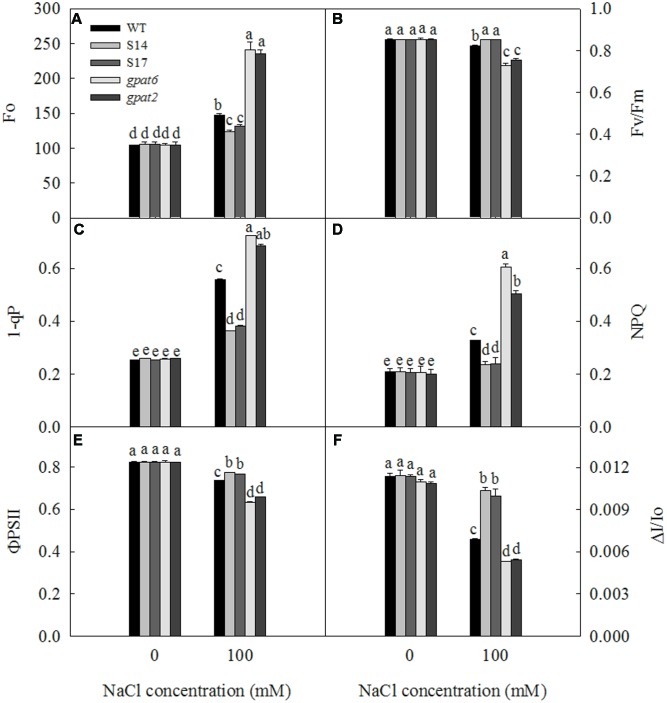
Effect of NaCl treatment on Fo **(A)**, Fv/Fm **(B)**, 1-qP **(C)**, NPQ **(D)**, ΦPSII **(E)**, and ΔI/Io **(F)** of WT, transgenic Arabidopsis plants and T-DNA mutant Arabidopsis strains. Data are presented as the means of six replicates ±*SD* (*n* = 6). For each column, different letters a, b, c, and d indicate a significant difference at *P* ≤ 0.05.

For almost all plants in the biosphere, photosynthesis is significant to their survival. With the increase of NaCl concentration to 200 mM, the net photosynthetic rate and the photochemical activity of chloroplas of *Arabidopsis thaliana* are decreased, resulting in the decline of photosynthesis (Zhao et al., 2007). As shown in **Figure 7**, under the condition where NaCl treatment was not performed, there were no significant differences in Fo, Fv/Fm, 1-qP, NPQ, ΦPSII and ΔI/Io among WT, overexpressed Arabidopsis strains and the mutant strains. However, when NaCl concentration was 100 mM, Fo, 1-qP, and NPQ in WT, overexpressed Arabidopsis strains and mutant Arabidopsis strains significantly increased compared to the controls; while Fv/Fm, ΦPSII and PSI all decreased under salt stress. Under salt treatment, Fo, 1-qP and NPQ of WT plants increased by 40.3, 120.2 and 58.2 %, respectively; Fo, 1-qP and NPQ of S14 increased by 17.4, 39.6 and 13.7%, respectively; Fo, 1-qP and NPQ of S17 increased by 24.2, 66.0 and 16.6%, respectively; Fo, 1-qP and NPQ of *gpat6* increased by 132.2, 180.4, and 191.7%, respectively; and Fo, 1-qP and NPQ of *gpat2* increased by 123.8, 164.6, and 150.4%, respectively. Fv/Fm of overexpressed Arabidopsis strains was not affected by salt stress. The Fv/Fm in WT, *gpat6* and *gpat2* decreased by 3.3, 14.6, and 11.5%, respectively. The ΦPSII activity in WT, S14, S17, *gpat6* and *gpat2* decreased by 10.7, 6.3, 6.9, 23.2, and 20.1%, respectively. The PSI oxidoreductive of WT, S14, S17, *gpat6* and *gpat2* decreased by 39.6, 8.8, 12.3, 51.7, and 50.2%, respectively.

### Fresh Weight and Dry Weight

**FIGURE 8:**
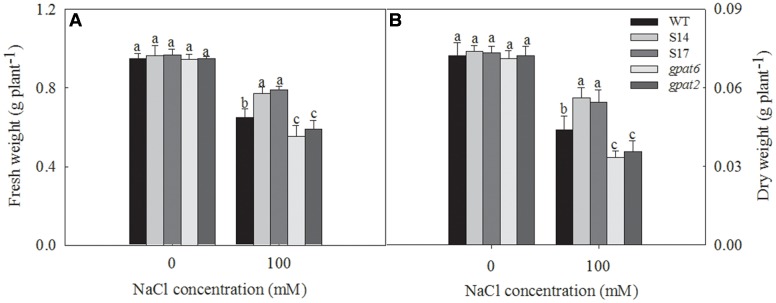
Biomass of fresh weight and dry weight of WT, transgenic Arabidopsis plants and T-DNA mutant Arabidopsis strains. The seedlings were treated with 0 and 100 mM NaCl for 14 days. Data are presented as the means of six replicates ±*SD* (*n* = 6). For each column, different letters a-n indicate a significant difference at *P* ≤ 0.05.

The growth of all Arabidopsis lines had no significant differences under the condition without NaCl treatment and were decreased under salt stress (**Figure 8**). Under 100 mM NaCl treatments, the fresh weight and dry weight of WT plants decreased by 31.6 and 38.9%, respectively. While they decreased by 19.7 and 24.2% in S14, 18.3 and 25.6% in S17, 41.4 and 53.1% in *gpat6* and 37.8 and 50.8% in *gpat2*, respectively.

## Discussion

*Suaeda salsa* is a typical saline-alkaline indicator plant. Low salt treatment does not inhibit the growth of *S. salsa*, but promotes its growth. GPAT catalyzes the initial and rate-limiting step of glycerolipid synthesis by transfering the acyl moiety to a glycerol-3-phosphate (G3P) molecule, thereby regulating the synthesis of triacylglycerol and all of the glycerophospholipids. The overexpression of Arabidopsis *AtGPAT* in tobacco can increase the content of unsaturated fatty acids in PG and increase resistance to chilling stress of tobacco (Murata et al., 1992). Furthermore, overexpression of Arabidopsis *AtGPAT* in rice can improve the photosynthetic rates and growth at low temperatures by increasing the degree of unsaturation of fatty acids in PG (Ariizumi et al., 2002). The overexpression of tomato *LeGPAT* can also increase chilling tolerance by increasing the degree of unsaturation fatty acids in PG (Sui et al., 2007c). In the present study, the cDNA of *SsGPAT* that encoded a protein of 388 amino acids (**Figure 1A**) was isolated from *S. salsa*. The amino acid sequence analysis revealed that it had the conservative structural functional domains of GPAT and the highest homology and closest relationship with spinach (**Figures 1B,C**). The expression of *SsGPAT* in the leaves of *S. salsa* under different salt concentrations revealed that the highest expression was at 300 mM NaCl treatment (**Figure 2**). This revealed that the expression of *SsGPAT* in *S. salsa* was induced by salt stress.

Salt stress can reduce the growth and development of plants, and inhibit cell division and expansion (Mahajan and Tuteja, 2005). In our study, by observing the germination phenotype of Arabidopsis, we found that the growth, germination rate and root length of WT, the overexpressed strains and the T-DNA insert mutants under 0, 50, 100 and 150 mM NaCl treatment were all inhibited. The suppression degree was significantly lower in the overexpressed strains and higher in the mutant strains than that in WT (**Figure 5**). This revealed that the overexpression of *SsGPAT* increased the salt tolerance of plants to a certain extent at the seed germination stage.

In the seedling stage, we found that the expression of the *SsGPAT* gene in the leaves of overexpressing lines increased with the increase in NaCl concentration and reached the highest level at 100 mM NaCl (**Figure 3D**). Furthermore, the fatty acids composition of PG, the chlorophyll content and the fluorescence parameters of WT and overexpressed strains were determined under 100 mM NaCl treatment (**Figures 6**, **7**). The hydrophobic lipid interior of the membrane can limit the transport of many ions and large molecules (Upchurch, 2008). Moreover, membrane integrity and the function are maintained by membrane structure and fluidity (Sui and Han, 2014). PG is an important component of photosynthetic membranes in protecting photosynthetic apparatuses (Domonkos et al., 2004). Fatty acids are major components of membranes and as such part of the mechanisms by which cellular processes are adapted to environmental constraints (Huang et al., 2017). GPAT was able to regulate the synthesis of PG and affect the synthesis of unsaturated fatty acids. Our previous study revealed that the overexperssion of *LeGPAT* in tomato increases unsaturated fatty acid content and alleviates the photoinhibition of PSII and PSI under chilling stress (Sui et al., 2007c). Sun et al. (2010) found that the increase of unsaturated fatty acid in PG content in thylakoid membrane lipids of tomato plants by overexpressing *LeGPAT* improves salt tolerance. The present experiment indicates that the overexpression of the *SsGPAT* gene in Arabidopsis enhanced salt tolerance by increasing the content of PG unsaturated fatty acid. In addition, the contents of PG unsaturated fatty acids in mutant *gpat6* and *gpat2* were lower than WT Arabidopsis. Higher unsaturated fatty acid content of PG in overexpressed plants (**Table 2**) alleviated the photoinhibiton of PSII and PSI (**Figures 7B,F**) and protected the membrane structure during salt stress. These results are in agreement with our previous report that *S. salsa* has high resistance to photoinhibition under salt stress and the increase of unsaturation of fatty acids enhances PSII tolerance to salt stress (Sui et al., 2010).

There are 10 members of GPAT family in Arabidopsis. *ATGPAT2* belongs to mitochondrial GPAT and *gpat2* is the mutant of *ATGPAT2*. *ATGPAT6* belongs to the endoplasmic reticulum GPAT, which is related to the formation of horny structures, chitin synthesis and the seed setting rate and is strongly expressed in inflorescence. GPAT6 is the mutant of *ATGPAT6*. At present, the study on the two genes is focused on the development and synthesis of oil and is less on salt tolerance. In the study of the three GPAT genes of *ATGPAT6*, *ATGPAT7*, and *ATGPAT9* in *Arabidopsis thaliana*, *ATGPAT6* and *ATGPAT7* can actively regulate the plants to response to salt stress (Hao, 2013). In our present study, it was found that seed germination and root length, chlorophyll content, PSII, and PSI activity, and PG fatty acid composition of *gpat6* and *gpat2* significantly decreased under salt stress compared to wild-type and transgenic Arabidopsis, indicating that *ATGPAT6* and *ATGPAT2* were related with salt resistance.

Chlorophyll is an important part of the light-harvesting complex (LHCII), which acts as antenna to capture light energy and transfer to the reaction center. Chlorophyll *a* molecules are important components in light-harvesting and the electron transfer reactions in photosynthesis, since these are the primary electron donors in the electron transport chain and thereby regulate the absorption, transition, and distribution of light energy (Sui and Han, 2014). Chlorophyll content reflects the photosynthesis strength of plants. The exposure of Arabidopsis to salt stress resulted in a progressive decline in chlorophyll *a* and *b* content (**Figures 6A,B**). Chlorophyll content decreased less in overexpressed strains and more in mutant strains than that in WT (**Figure 6**). The less decrease of Chl content in overexpressed strains means less effect on light absorption, transition and distribution. Higher chlorophyll content inevitably results in the higher photochemical efficiency of PSII in overexpressed strains (**Figure 7**), which is consistent with our previous study (Sui and Han, 2014). The Chl *a/b* ratio reflects the stacking extent of the thylakoid membrane, that is, the proportion of stacked thylakoid membrane (Aro et al., 1993). The chl *a/b* ratio decreased less in overexpressed strains and more in the mutant strains relative to WT under 100 mM NaCl treatment (**Figure 6C**). This revealed that light energy harvested by LHCII was absorbed more in overexpression strains and less in the mutant strains relative to WT, which result in the different degrees of photoinhibition of PSII. This was demonstrated by the value of Fv/Fm (**Figure 7B**).

Chlorophyll fluorescence can reflect the absorption of light energy, photochemical reaction, electron transfer and the establishment of the proton gradient, and almost all photosynthetic process changes can be reflected by chlorophyll fluorescence. The changes of Fo depends on the dominant factor between energy dissipation and the inactivation or damage of PSII. The inactivation or the damage of PSII causes the increase of Fo (Xu and Wu, 1996). In the present study, we revealed that Fo increased in WT, overexpressed strains and mutant strains (**Figure 7A**). Under 100 mM NaCl treatment, however, the Fo increase in the overexpressed strains was less than that in WT and the mutant strains. The extent of PSII photoinhibition is closely correlated with the redox state of Q_A_ under a range of stress condition (Havaux et al., 1991; Sui, 2015). The relative redox state of Q_A_ *in vivo* can be estimated as 1-qP (Qin et al., 2011). Our results revealed that 1-qP in WT, overexpressed strains and mutant strains increased under 100 mM NaCl treatment, which was even greater in the mutant strains (**Figure 7C**). This suggests that the extent of Q_A_ reduction is more severe in GPAT deletion strains. The xanthophyll cycle dependent NPQ mechanism constitutes an important protective response to prevent the over-reduction of Q_A_ (Demmig-Adams and Adams, 1996). The increase of 1-qP was accompanied by the marked increase in NPQ (**Figure 7D**). However, this increase was lowest in overexpressed strains. This suggests that the extent of PSII photoinhibition correlated with the redox state of Q_A_ is alleviated by the overexpression of *SsGPAT*. The ΦPSII decreased less in overexpressed strains under salt stress, suggesting that PSII of overexpressed strains was rather tolerant to salt stress and had effective mechanisms to protect photosystem from salt stress. If excess energy could not be dissipated and CO_2_ assimilation is blocked under salt stress, PSI reaction centers will reduce to produce triplet P700. Domonkos et al. (2008) demonstrates that the decrease of PSI activity is related with the monomerization of the trimer of PSI in the mutant of *Synechocystis* PCC6803. They report that the depletion of PG results in the degradation of PSI trimers and the concomitant accumulation of monomer PSI. This suggests that PG plays an important role not only in PSII but also in PSI, perhaps in the assembly of the PSI core complex (Jordan et al., 2001; Sakurai et al., 2003; Sui et al., 2007c). In the present study, we revealed that PSI oxidoreductive activity (ΔI/Io) decreased in WT, overexpressed strains and the mutant strains, but this decrease is much less in overexpressed strains than that in WT and mutant strains (**Figure 7F**). Furthemore, the serious decline in mutant strains might be attributed not only to the limitation of electron acceptors, but also probably to the damage of PSI components. In this study, we also observed significant reductions in fresh weight and dry weight of all Arabidopsis lines under 100 mM NaCl treatment. But the decrease was less in overexpressed strains and more in mutant strains than that in WT (**Figure 8**). The trend of biomass in WT, overexpressed strains and the mutant strains is consistent with chlorophyll content, Fv/Fm and ΦPSII under salt stress. This suggests that higher Chl content (**Figure 6**) results in higher photochemical efficiency of PSII (**Figure 7**), which inevitably leads to higher production in overexpressed strains (**Figure 8**).

## Conclusion

We demonstrated that *S. salsa SsGPAT* transcription was activated by high salt. The overexpression of *SsGPAT* in Arabidopsis increased *cis*-unsaturated fatty acid levels in PG. The increase of PG *cis*-unsaturated fatty acids protected the photosynthesis apparatus and maintained membrane function against salt stress by alleviating the photoinhibition of PSII and PSI. However, the mechanism of unsaturated fatty acid for increasing salt tolerance should be addressed in future studies.

## Author Contributions

NS and ST wrote this manuscript; ST, WW, and MW performed experiments; ST and WW collected data and carried out all analyses; NS and HF conceptualized the idea and revised the manuscript.

## Conflict of Interest Statement

The authors declare that the research was conducted in the absence of any commercial or financial relationships that could be construed as a potential conflict of interest.

## References

[B1] Allakhverdiev S. I. Kinoshita M. Inaba M. Suzuki I. Murata N. (2001). Unsaturated fatty acids in membrane lipids protect the photo-synthetic machinery against salt-induced damage in *Synechococcus*. *Plant Physiol.* 125 1842–1853. 10.1104/pp.125.4.1842 11299364 PMC88840

[B2] Allakhverdiev S. I. Sakamoto A. Nishiyama Y. Inaba M. Murata N. (2000). Ionic and osmotic effects of NaCl-induced inactivation of photosystems I and II in *Synechococcus* sp. *Plant Physiol.* 123 1047–1056. 10.1104/pp.123.3.1047 10889254 PMC59068

[B3] Ariizumi T. Kishitani S. Inatsugi R. Nishida I. Murata N. Toriyama K. (2002). An increase in unsaturation of fatty acids in phosphatidyl-glycerol from leaves improves the rates of photosynthesis and growth at low temperatures in transgenic rice seedlings. *Plant Cell Physiol.* 43 751–758. 10.1093/pcp/pcf087 12154137

[B4] Aro E. Virgin I. Andersson B. (1993). Photoinhibition of photosystem II inactivation, protein damage and turnover. *Biochim. Biophys. Acta* 1143 113–134. 10.1016/0005-2728(93)90134-2 8318516

[B5] Baker N. R. (1991). A possible role for photosystem II in environmental perturbations of photosynthesis. *Physiol. Plant.* 81 563–570. 10.1111/j.1399-3054.1991.tb05101.x

[B6] Chen Z. Q. Xu C. H. Chen M. J. Xu L. Wang K. F. Lin S. Q. (1994). Effect of chilling acclimation on thylakoid membrane protein of wheat. *Acta Bot. Sin.* 36 423–429.

[B7] Cheng S. Yang Z. Wang M. J. Song J. Sui N. Fan H. (2014). Salinity improves chilling resistance in *Suaeda salsa*. *Acta Physiol. Plant.* 36 1823–1830. 10.1007/s11738-014-1555-3

[B8] Cooke D. T. Burden R. S. (1990). Lipid modulation of plasma membrane-bound ATPases. *Physiol. Plant.* 78 153–159. 10.1111/j.1399-3054.1990.tb08730.x

[B9] Dakhma W. S. Zarrouk M. Cherif A. (1995). Effects of drought-stress on lipids in rape leaves. *Phytochemistry* 40 1383–1386. 10.1016/0031-9422(95)00459-K

[B10] Demmig-Adams B. Adams W. W. (1996). The role of xanthophyll cycle carotenoids in the protection of photosynthesis. *Trends Plant Sci.* 1 21–26. 10.1016/S1360-1385(96)80019-7

[B11] Deuticke B. Haest C. (1987). Lipid modulation of transport proteins in vertebrate cell membranes. *Annu. Rev. Physiol.* 49 221–235. 10.1146/annurev.ph.49.030187.001253 2436568

[B12] Domonkos I. Laczkó-Dobos H. Gombos Z. (2008). Lipid-assisted protein–protein interactions that support photosynthetic and other cellular activities. *Prog. Lipid Res.* 47 422–435. 10.1016/j.plipres.2008.05.003 18590767

[B13] Domonkos I. Malec P. Sallai A. Kovacs L. Itoh K. Shen G. (2004). Phosphatidylglycerol is essential for oligomerization of photosystem I reaction center. *Plant Physiol.* 134 1471–1478. 10.1104/pp.103.037754 15064373 PMC419823

[B14] Flowers T. J. Colmer T. D. (2008). Salinity tolerance in halophytes. *New Phytol.* 179 945–963. 10.1111/j.1469-8137.2008.02531.x 18565144

[B15] Gidda S. K. Shockey J. M. Rothstein S. J. Dye R. J. M. Mullen R. T. (2009). *Arabidopsis thaliana* GPAT8 and GPAT9 are localized to the ER and possess distinct ER retrieval signals: functional divergence of the dilysine ER retrieval motif in plant cells. *Plant Physiol. Biochem.* 47 867–879. 10.1016/j.plaphy.2009.05.008 19539490

[B16] Hao J. F. (2013). *The Functions of Three Genes of Glycerol-3-phossphate Acyl Transferase (GPAT6 7 9) in Regulation of the Seed-Oil Content and the Salt Tolerance in Arabidopsis*. Master’s thesis, Nanjing Agricultural College, Nanjing.

[B17] Hasegawa P. M. Bressan R. A. Zhu J. Bohnert H. J. (2000). Plant cellular and molecular responses to high salinity. *Annu. Rev. Plant Biol.* 51 463–499. 10.1146/annurev.arplant.51.1.463 15012199

[B18] Havaux M. Strasser R. J. Greppin H. (1991). A theoretical and experimental analysis of the qp and qn coefficients of chlorophyll fluorescence quenching and their relation to photochemical and nonphotochemical events. *Photosynth. Res.* 1 41–55. 10.1007/BF00029975 24414444

[B19] Huang J. Xue C. Wang H. Wang L. Schmidt W. Shen R. (2017). Genes of ACYL CARRIER PROTEIN family show different expression profiles and overexpression of ACYL CARRIER PROTEIN 5 modulates fatty acid composition and enhances salt stress tolerance in Arabidopsis. *Front. Plant Sci.* 8:987 10.3389/fpls.2017.00987 PMC5463277 28642782

[B20] Huang W. Li Z. G. Qiao H. L. Li C. Z. Liu X. J. (2008). Interactive effect of sodium chloride and drought on growth and osmotica of *Suaeda salsa*. *Chin. J. Eco Agric.* 16 173–178. 10.3724/SP.J.1011.2008.00173

[B21] Ioannidis N. E. Sfichi-Duke L. Kotzabasis K. (2006). Putrescine stimulates chemiosmotic ATP synthesis. *Biochim. Biophys. Acta* 1757 821–828. 10.1016/j.bbabio.2006.05.034 16828052

[B22] Jordan P. Formme P. Witt H. T. Klukas O. Saenger W. Krauss N. (2001). Three-dimensional structure of cyanobacterial photosystem I at 2.5 Å resolution. *Nature* 411 909–917. 10.1038/35082000 11418848

[B23] Joyard J. Ferro M. Masselon C. Seigneurin-Berny D. Salvi D. Garin J. (2010). Chloroplast proteomics highlights the subcellular compartmentation of lipid metabolism. *Prog. Lipid Res.* 49 128–158. 10.1016/j.plipres.2009.10.003 19879895

[B24] Kooten O. Snel J. F. (1990). The use of chlorophyll fluorescence nomenclature in plant stress physiology. *Photosynth. Res.* 25 147–150. 10.1007/BF00033156 24420345

[B25] Li X. G. Meng Q. W. Jiang G. Q. Zou Q. (2003). The susceptibility of cucumber and sweet pepper to chilling under low irradiance is related to energy dissipation and water–water cycle. *Photosynthetica* 41 259–265. 10.1023/B:PHOT.0000011959.30746.c0

[B26] Liu M. Z. Michael B. J. Henry J. S. (2013). Efficient planar heterojunction perovskite solar cells by vapour deposition. *Nature* 501 395–398. 10.1038/nature12509 24025775

[B27] Liu X. Li B. Yang J. Sui N. Yang X. Meng Q. (2008). Overexpression of tomato chloroplast omega-3 fatty acid desaturase gene alleviates the photoinhibition of photosystems II and I under chilling stress. *Photosynthetica* 46 185–192. 10.1007/s11099-008-0030-z

[B28] Lu C. M. Qiu N. W. Lu Q. Wang B. S. Kuang T. Y. (2002). Does salt stress lead to increased susceptibility of photosystem II to photoinhibition and changes in photosynthetic pigment composition in halophyte *Suaeda salsa* grown outdoors. *Plant Sci.* 5 1063–1068. 10.1016/S0168-9452(02)00281-9

[B29] Mahajan S. Tuteja N. (2005). Cold, Salinity and drought stresses: an overview. *Arch. Biochem. Biophys.* 444 139–158. 10.1016/j.abb.2005.10.018 16309626

[B30] Matos M. C. Campos P. S. Ramalho J. C. Medeira M. C. Maia M. I Semedo J. M. (2002). Photosynthetic activity and cellular integrity of the Andean legume *Pachyrhizus ahipa* (Wedd.) Parodi under heat and water stress. *Photosynthetica* 40 493–501. 10.1023/A:1024331414564

[B31] Mikami K. Murata N. (2003). Membrane fluidity and the perception of environmental signals in cyanobacteria and plants. *Prog. Lipid Res.* 42 527–543. 10.1016/S0163-7827(03)00036-5 14559070

[B32] Mittova V. Guy M. Tal M. Volokita M. (2004). Salinity up-regulates the antioxidative system in root mitochondria and peroxisomes of the wild salt-tolerant tomato species *Lycopersicon pennellii*. *J. Exp. Bot.* 55 1105–1113. 10.1093/jxb/erh113 15047761

[B33] Munns R. Tester M. (2008). Mechanisms of salinity tolerance. *Annu. Rev. Plant Biol.* 59 651–681. 10.1146/annurev.arplant.59.032607.092911 18444910

[B34] Murata N. Ishizaki-Nishizawa O. Higashi S. Hayashi H. Tasaka Y. Nishida I. (1992). Genetically engineered alteration in the chilling sensitivity of plants. *Nature* 356 710–713. 10.1038/356710a0

[B35] Olsson M. (1995). Alterations in lipid composition, lipid peroxidation and anti-oxidative protection during senescence in drought stressed plants and non-drought stressed plants of *Pisum sativum*. *Plant Physiol. Biochem.* 33 547–553.

[B36] Payá-Milans M. Aznar-Moreno J. A. Balbuena T. S. Haslam R. P. Gidda S. K. Pérez-Hormaeche J. (2016). Sunflower *HaGPAT9-1* is the predominant GPAT during seed development. *Plant Sci.* 252 42–52. 10.1016/j.plantsci.2016.07.002 27717477

[B37] Qin L. Q. Li L. Bi C. Zhang Y. L. Wan S. B. Meng J. J. (2011). Damaging mechanisms of chillingand salt stress to *Arachis hypogaea* L. leaves. *Photosynthetica* 49 37–42. 10.1007/s11099-011-0005-3

[B38] Roughan P. G. Slack C. R. (1982). Cellular organization of glycerolipid metabolism. *Annu. Rev. Plant Physiol.* 33 97–132. 10.1146/annurev.pp.33.060182.000525

[B39] Sakurai I. Hagio M. Gombos Z. Tyystjärvi T. Paakkarinen V. Aro E. M. (2003). Requirement of phosphatidylglycerol for maintenance of photosynthetic machinery. *Plant Physiol.* 133 1376–1384. 10.1104/pp.103.026955 14551333 PMC281632

[B40] Schreiber U. B. W. N. Bilger W. Neubauer C. (1995). “Chlorophyll fluorescence as a nonintrusive indicator for rapid assessment of *in vivo* photosynthesis,” in *Ecophysiology of Photosynthesis* Vol. 100 eds Schulze E. D. Caldwell M. M. (Berlin: Springer), 49–70.

[B41] Schuler I. Milon A. Nakatani Y. Ourisson G. Albrecht A. Benveniste P. (1991). Differential effects of plant sterols on water permeability and on acyl chain ordering of soybean phosphatidylcholine bilayers. *Proc. Natl. Acad. Sci. U.S.A.* 88 6926–6930. 10.1073/pnas.88.16.6926 11607205 PMC52206

[B42] Sharkey T. D. Badger M. R. (1982). Effect of water stress on photosynthetic electron transport, photophosphory-lation and metabolite levels of xanthium strumarium mesophyll cells. *Planta* 156 199–206. 10.1007/BF00393725 24272466

[B43] Shu S. Sun J. Guo S. R. Li J. Liu C. J. Wang C. Y. (2010). Effects of exogenous putrescine on PSII photochemistry and ion distribution seedlings under salt stress. *Acta Hortic. Sin.* 37 1065–1107.

[B44] Siegenthaler P. A. Eichenberger W. (1984). “Structure, function, and metabolism of plant lipids,” in *Proceedings of the 6th International Symposium on the Structure, Function, and Metabolism of Plant Lipids Held in Neuchâtel* Vol. 9 (Switzerland: Elsevier Science Publishers), 16–20.

[B45] Song J. (2009). Root morphology is related to the phenotypic variation in water logging tolerance of two populations of *Suaeda salsa* under salinity. *Plant Soil* 324 231–240. 10.1007/s11104-009-9949-5

[B46] Song J. Chen M. Feng G. Jia Y. H. Wang B. S. Zhang F. S. (2009). Effect of salinity on growth, ion accumulation and the roles of ions in osmotic adjustment of two populations of *Suaeda salsa*. *Plant Soil* 314 133–141. 10.1007/s11104-008-9712-3

[B47] Song J. Fan H. Zhao Y. Y. Jia Y. H. Du X. H. Wang B. S. (2008). Effect of salinity on germination, seedling emergence, seedling growth and ion accumulation of a euhalophyte *Suaeda salsa* in an intertidal zone and on saline inland. *Aquat. Bot.* 88 331–337. 10.1016/j.aquabot.2007.11.004

[B48] Stepien P. Klobus G. (2005). Antioxidant defense in the leaves of C3 and C4 plants under salinity stress. *Physiol. Plant.* 125 31–40. 10.1111/j.1399-3054.2005.00534.x

[B49] Sui N. (2015). Photoinhibition of *Suaeda salsa* to chilling stress is related to energy dissipation and water-water cycle. *Photosynthetica* 53 207–212. 10.1007/s11099-015-0080-y

[B50] Sui N. Han G. L. (2014). Salt-induced photoinhibition of PSII is alleviated in halophyte Thellungiella halophila by increases of unsaturated fatty acids in membrane lipids. *Acta Physiol. Plant* 36 983–992. 10.1007/s11738-013-1477-5

[B51] Sui N. Li M. Li K. Song J. Wang B. S. (2010). Increase in unsaturated fatty acids in membrane lipids of *Suaeda salsa* L. enhances protection of photosystem II under high salinity. *Photosynthetica* 48 623–629. 10.1007/s11099-010-0080-x

[B52] Sui N. Li M. Liu X. Y. Wang N. Fang W. Meng Q. W. (2007a). Response of xanthophyll cycle and chloroplastic antioxidant enzymes to chilling stress in tomato over-expressing glycerol-3-phosphate acyltransferase gene. *Photosynthetica* 45 447–454. 10.1007/s11099-007-0074-5

[B53] Sui N. Li M. Shu D. F. Zhao S. J. Meng Q. W. (2007b). Antisense-mediated depletion of tomato chloroplast glycerol-3-phosphate acyltransferase affects male fertility and increases thermal tolerance. *Physiol. Plant.* 130 301–314. 10.1111/j.1399-3054.2007.00907.x

[B54] Sui N. Li M. Zhao S. Li F. Liang H. Meng Q. (2007c). Overexpression of glycerol-3-phosphate acyltransferase gene improves chilling tolerance in tomato. *Planta* 226 1097–1108. 17541789 10.1007/s00425-007-0554-7

[B55] Sun Y. L. Li F. Sui N. Sun X. L. Zhao S. J. Meng Q. W. (2010). The increase in unsaturation of fatty acids of phosphatidylglycerol in thylakoid membrane enhanced salt tolerance in tomato. *Photosynthetica* 48 400–408. 10.1007/s11099-010-0052-1

[B56] Upchurch R. G. (2008). Fatty acid unsaturation, mobilization, and regulation in the response of plants to stress. *Biotechnol. Lett.* 30 967–977. 10.1007/s10529-008-9639-z 18227974

[B57] Wada H. Murata N. (1998). “Membrane lipids in cyanobacteria,” in *Lipids in Photosynthesis: Structure, Function and Genetics* Vol. 6 eds Paul-André S. Norio M. (Springer: Dordrecht), 65–81.

[B58] Wei A. L. Chen Y. Z. (2000). Effect of IAA on soybean seedling’s membrance injury and salt resistance. *Acta Bot. Boreali Occidentalia Sin.* 20 410–414.

[B59] Xu D. Q. Wu S. (1996). Three phases of dark-recovery course from photoinhibition resolved by the chlorophyll fluorescence analysis in soybean leaves under field conditions. *Photosynthetica* 32 417–423.

[B60] Xu Y. Siegenthaler P. (1997). Low temperature treatments induce an increase in the relative content of both linolenic and λ3-hexadecenoic acids in thylakoid membrane phosphatidylglycerol of squash cotyledons. *Plant Cell Physiol.* 38 611–618. 10.1093/oxfordjournals.pcp.a029211

[B61] Zhang J. T. Zhu J. Q. Zhu Q. Liu H. Gao X. S. Zhang H. X. (2009). Fatty acid desaturase-6 (Fad6) is required for salt tolerance in *Arabidopsis thaliana*. *Biochem. Biophys. Res. Commun.* 390 469–474. 10.1016/j.bbrc.2009.09.095 19799856

[B62] Zhang R. H. Guo S. R. Li J. (2006). Effects of salt stress on root activity and chlorophyll content of cucumber. *J. Chang Jiang Veget* 2 47–49.

[B63] Zhang X. Henriques R. Lin S. S. Niu Q. W. Chua N. H. (2006). Agrobacterium-mediated transformation of *Arabidopsis thaliana* using the floral dip method. *Nat. Protoc.* 1 641–646. 10.1038/nprot.2006.97 17406292

[B64] Zhao K. F. Fan H. Jiang X. Y. Song J. (2002). Improvement and utilization of saline soil by planting halophytes. *Chin. J. Appl. Environ. Biol.* 8 31–35.

[B65] Zhao X. Wu Y. X. Zhao M. J. He J. X. (2007). Effects of NaCl Stress on Photosynthesis of *Thellungiella salsuginea* and *Arabidopsis thaliana*. *Bull. Bot.* 24 154–160.

[B66] Zheng Z. Xia Q. Dauk M. Shen W. Selvarai G. Zou J. (2003). Arabidopsis AtGPAT1, a member of the membrane-bound glycerol-3-phosphate acyltransferase gene family, is essential for tapetum differentiation and male fertility. *Plant Cell* 15 1872–1887. 10.1105/tpc.012427 12897259 PMC167176

[B67] Zhu J. K. (2001). Plant salt tolerance. *Trends Plant Sci.* 6 66–71. 10.1016/S1360-1385(00)01838-0 11173290

[B68] Zhu J. K. (2016). Abiotic stress signaling and responses in plants. *Cell* 167 312–324. 10.1016/j.cell.2016.08.029 PMC5104190 27716505

[B69] Zhu Z. H. Hu R. H. (1996). Effects of salt stress on seed germination of different varieties wheat. *China Seeds* 4 25–29.

